# The Technology Transfer from Europe to China in the 17th–18th Centuries: Non-Invasive On-Site XRF and Raman Analyses of Chinese Qing Dynasty Enameled Masterpieces Made Using European Ingredients/Recipes

**DOI:** 10.3390/ma14237434

**Published:** 2021-12-03

**Authors:** Philippe Colomban, Michele Gironda, Divine Vangu, Burcu Kırmızı, Bing Zhao, Vincent Cochet

**Affiliations:** 1 MONARIS (UMR8233), Sorbonne Université, Campus P. et M. Curie, CNRS, 4 Place Jussieu, 75005 Paris, France; divine.vangu@etu.sorbonne-universite.fr; 2 XGLab S.R.L—Bruker, 23 Via Conte Rosso, 20134 Milan, Italy; michele.gironda@bruker.com; 3 Department of Conservation and Restoration of Cultural Property, Faculty of Architecture, Yıldız Technical University, Yıldız Yerleşkesi B Blok, Beşiktaş, Istanbul 34349, Turkey; kirmizi@yildiz.edu.tr; 4 CNRS, CRCAO, UMR8155, Collège de France, 75005 Paris, France; bing.zhao@college-de-france.fr; 5 Musée National du Château de Fontainebleau, Place Charles de Gaulle, 77300 Fontainebleau, France; vincent.cochet@chateaudefontainebleau.fr

**Keywords:** painted enamels, *cloisonné*, gold alloy, blue, yellow, green, white, China, Qing Dynasty, spectroscopy, composition

## Abstract

Two masterpieces of the Qing Dynasty (1644–1912 CE), one in gilded brass (incense burner) decorated with *cloisonné* enamels stylistically attributed to the end of the Kangxi Emperor’s reign, the other in gold (ewer offered by Napoleon III to the Empress as a birthday present), decorated with both *cloisonné* and painted enamels bearing the mark of the Qianlong Emperor, were non-invasively studied by optical microscopy, Raman microspectroscopy and X-ray microfluorescence spectroscopy (point measurements and mapping) implemented on-site with mobile instruments. The elemental compositions of the metal substrates and enamels are compared. XRF point measurements and mappings support the identification of the coloring phases and elements obtained by Raman microspectroscopy. Attention was paid to the white (opacifier), blue, yellow, green, and red areas. The demonstration of arsenic-based phases (e.g., lead arsenate apatite) in the blue areas of the ewer, free of manganese, proves the use of cobalt imported from Europe. The high level of potassium confirms the use of smalt as the cobalt source. On the other hand, the significant manganese level indicates the use of Asian cobalt ores for the enamels of the incense burner. The very limited use of the lead pyrochlore pigment (European *Naples yellow* recipes) in the yellow and soft green *cloisonné* enamels of the Kangxi incense burner, as well as the use of traditional Chinese recipes for other colors (white, turquoise, dark green, red), reinforces the pioneering character of this object in technical terms at the 17th–18th century turn. The low level of lead in the *cloisonné* enamels of the incense burner may also be related to the use of European recipes. On the contrary, the Qianlong ewer displays all the enameling techniques imported from Europe to obtain a painted decoration of exceptional quality with the use of complex lead pyrochlore pigments, with or without addition of zinc, as well as cassiterite opacifier.

## 1. Aim

The attention paid to the construction of a global history [1,2,3] not centered on Europe or Asia has led to the symmetrical study of exchanges of all kinds between Europe and the Far East for several years, especially regarding fine arts [4,5,6,7,8,9,10,11]. The pioneering role of Chinese potters in the development of porcelain production is well known [4,5,6,7,8,9,10,11,12,13,14], and the enthusiasm of European elites for these ceramics from the well-established importation of blue-and-white porcelain by Portuguese then Dutch and English sea trade in the 16th and 17th centuries has been largely studied [15,16,17]. The role of the Jesuits living in Japan [18,19,20,21,22] and China [23,24,25,26,27] in the circulation of European science related to astronomy [22], mathematics [23], time measurement [21] and painting [26,27,28] is also rather well established. The role of Jesuits in the transfer of enameling technologies is less documented and only studied for a few years by research on the Imperial Palace archives [29] and the correspondence of the Society of Jesus [21,22,23] or diplomatic issues [30,31,32,33]. Our objective is to seek in the material of the ‘Chinese’ objects themselves the evidence of the use of European ingredients or recipes in the enameling procedures, similar to what was done for the first porcelains of Arita (Japan) in the late 16th century [18,19,20]. If shards are available, which is not the case most of the time for outstanding artefacts, it is possible to use micro-destructive methods [4,34,35,36] and many types of scientific analyses can be carried out in the laboratory using a variety of instruments. However, the analytical methodology for the intact invaluable objects is more restricted and difficult since it must be carried out in a perfectly non-invasive way, without any sampling and contact. The rarity and great value of the objects studied as well as their possible large size make their transfer to the laboratory facilities very expensive and impractical, inducing the necessity of on-site analysis in the exhibition halls or museum reserves with the use of mobile analytical instruments.

For several years, we have been developing procedures and models allowing the implementation and interpretation of the results obtained by mobile Raman microscopy and X-ray fluorescence devices [37,38,39,40,41,42,43,44,45,46,47,48,49,50,51], specifically for the identification of coloring agents and silicate matrices of the glassy materials such as the enamels. Analyses were thus carried out on the collections of enameled objects (glass, metal or porcelain) produced in France and China between the 17th and the 19th century [10,36,38,39,44,45,46,52,53], allowing us to identify the objects most representative of the technological change for the period mentioned.

We present here the in-depth non-invasive analysis of two masterpieces from the collections of the National Museum of the Château de Fontainebleau (Chinese Museum), particularly constituted by the Emperor Napoleon III and his wife the Empress Eugenie in the second part of the 19th century [54,55,56]. The two objects studied have been selected as representative of the beginning of the introduction of European recipes (a gilded brass incense burner decorated with *cloisonné* enamels and stylistically attributed to the end of the Kangxi’s reign) and of the achievement of the mastering of this new technology (a gold ewer belonging to a set comprising a pair of ewers and a large gold basin with the Qianlong’s reign mark decorated with both painted and *cloisonné* enamels), respectively, among the hundred Chinese outstanding artefacts studied [10,42,52,53]. Other studies reported in the literature are limited in scope and have been performed on more common objects or sherds [34,35].

## 2. Materials and Methods

### 2.1. Portable X-ray Fluorescence Spectroscopy (pXRF)

X-Ray Fluorescence analysis was performed on-site using a portable ELIO instrument (ELIO, XGLab Bruker, Milan, Italy). The set-up includes a miniature X-ray tube system with a Rh anode (max voltage of 50 kV, max current of 0.2 mA, and a 1 mm collimator), and a large area Silicon Drift Detector (SDD, 50 mm^2^ active area) (ELIO, XGLab Bruker, Milan, Italy) with energy resolution of <140 eV for Mn Kα, an energy range of detection from 1 keV to 40 keV, and a maximum count rate of 5.6 × 10^5^ cps. Measurements were carried out in the point mode with an acquisition time of 40 s, using a tube voltage of 40 kV and current of 100 μA. No filter was used between the X-Ray tube and the sample. Three measurements were made for each colored area. The working distance (distance between the sample and detector) during analysis was around 15 mm, the distance between the instrument front and the artefact being about 10 mm. Spectral signals were obtained with the optimization of the signal-to-noise ratio (SNR) by selecting the set-up parameters chosen. Information thickness during analysis of the enamel is estimated to be close to 4 µm at Si K_α_, 130 µm at Cu K_α_, 220 µm at Au L_α_, and 2.5 mm at Sn K_α_. Within the resolution of the pXRF instrument, the Fe K_β_ peak and the Co K_α_ peak are located in the same energy range. To identify the presence of Co in enamels (except when cobalt is present in traces) we can use the information obtained looking at the Fe K_α_/Fe K_β_ ratios. In the absence of cobalt, the relative intensity between Fe K_α_ and Fe K_β_ peaks is about 6/1. Cobalt is then obvious if the superimposed peaks of Co K_α_ and Fe K_β_ exhibit a stronger intensity than that expected from the above ratio. Calculation of the local composition is also useful to detect cobalt, but the calculated ‘composition’ is not valid and only comparison of the counts is reliable.

XRF mapping was performed on different areas of the objects. The ELIO instrument is designed to move in front of the object measuring elemental maps up to 10 cm × 10 cm. The map is acquired in the raster scan mode with a pixel measurement time which can be configured by the user. The pixel size can be adjusted by selecting the motor step, but the spot resolution (around 1 mm) influences the spatial resolution. Good flatness of the area analyzed is required and it should be perpendicular to the axis of the instrument. Different map sizes were acquired selecting surface areas that were sufficiently flat for the tolerance of the system (plus/minus 2 mm in distance tolerance). The pixel acquisition time was set to 1 s per pixel with a pixel size of 1 mm. The ELIO software (2021, ELIO XGLab Bruker, Milan, Italy) allows the visualization of the different elements by showing the differences in intensity over the surface of a selected region of interest (ROI) in the spectrum. For element deconvolution and more advanced elaborations, the Bruker ESPRIT Reveal software (2011, Bruker AXS, Berlin, Germany) was used.

The data obtained were processed using the factory provided data reduction software, which enables automatic peak recognition supported by manual peak selection and checking. The software also enables curve fitting based on chosen elements to ensure a match between the measured spectra and theoretically predicted spectra calculated from fundamental parameters (FP). Semi-quantitative elemental compositions were also calculated for the elements of interest using FP by the instrument software when the sample thickness can be considered to fulfil the infinite thickness criteria and the material is homogeneous at the scale of the analyzed volume.

### 2.2. Raman Microspectroscopy

Raman analyses were carried out at the museum exhibition room with a mobile HE532 Raman set-up (HORIBA Scientific Jobin-Yvon, Longjumeau, France) as extensively described in the references [45,46,52,53]. For each colored area in the objects, at least three Raman spectra were recorded to obtain the representativeness of the collected data on a statistical basis. The reliability of the Raman spectrum starts above 80 cm^−1^ but a flat spectral background is only obtained over 500 cm^−1^. A 200× microscope objective (~13 mm long working distance) Mitutoyo Corp., Kawasaki, Japan) was used for the analysis of the ewer while 200× and 50× (17 mm long working distance) Nikon France SAS, Champigny-sur-Marne, France) objectives were used for the study of the incense burner. These objectives provide small, focused beams (surface spot waist ~0.5 µm and 2 µm; in-depth ~2 µm and 5–10 µm, respectively, the values varying with the color), perpendicular to the sample surface, which allow the recording of spectra not/poorly contaminated by the sub-layers and/or the environment. The 200× objective is more efficient for the analysis of the individual pigment grains embedded in the glassy matrix since it provides a very small laser spot which requires a very precise focus. Thus, it allows to obtain spectra with less background than those obtained using less sophisticated objectives with lower magnification. In fact, the shape of the spectral background gives information about the color of the analyzed spot: flat for a blue area, decreasing above ~500 cm^−1^ for a red area and increasing for yellow or green areas (see further). Obviously, the power of illumination at the sample should be minimal (1 mW or less) for black or dark colored areas due to the absorption of light, although up to 10 mW is required for light colored or colorless areas of the enamels. A linear segment baseline was subtracted using Labspec^®^ (HORIBA) software (5.25.15, 2007, HORIBA Jobin-Yvon, Longjumeau, France [36] and then the different components of the Raman spectrum were identified using Peakfitting Origin^®^ (6.0, 1999, Microcalc Inc., Northampton, MA, USA) software. Lorentzian and Gaussian shapes were used for narrow and broad components, respectively.

## 3. Objects Studied

Imperial workshops were established at the Forbidden City in Beijing to satisfy the demand of Kangxi Emperor that ‘new’ objects similar to those given as presents by the Jesuits and Emissary of Louis XIV could be locally produced. The first imperial workshop (Zaobanchu, Office of Manufacture) opened in 1693 to manufacture *cloisonné* enamels. It is assumed that preparation of painted enamels had started with the opening of a glass workshop in 1696. This unit was headed by the German Jesuit Kilian Stumpf who is considered to have introduced the European glassmaking techniques into China as a scientist and glassblower. A dedicated enameling workshop then opened in 1716 [6,29]. At the same time, or before, another production center of painted enamels was established in Guanzhou (Custom district). Indeed, many artefacts were commissioned by the Court as tributes [11]. Technological development of the overglaze porcelain palette expanded significantly at the end of Kangxi reign to produce Famille rose, Famille verte and Famille noire porcelains [4].

### 3.1. The Origin of Objects

**Figure 1:**
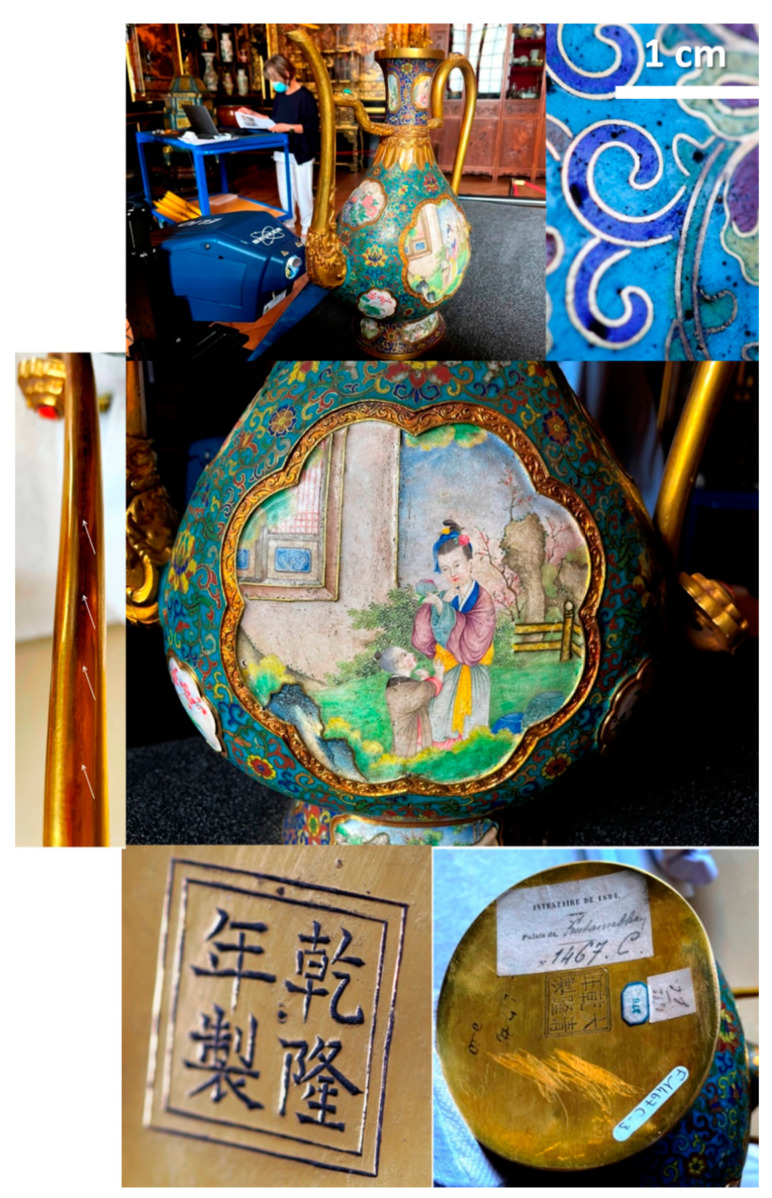
On-site pXRF analysis of the enameled ewer (F1467C) and details of the décor (*cloisonné* and painted enamels); the bottom face exhibits an engraved Qianlong reign mark (bottom left, basin: *Qian long nian zhi*; right, ewer; *Da qing Qian long nian zhi*). Note the welding traces along the spout (arrows).

**Figure 2:**
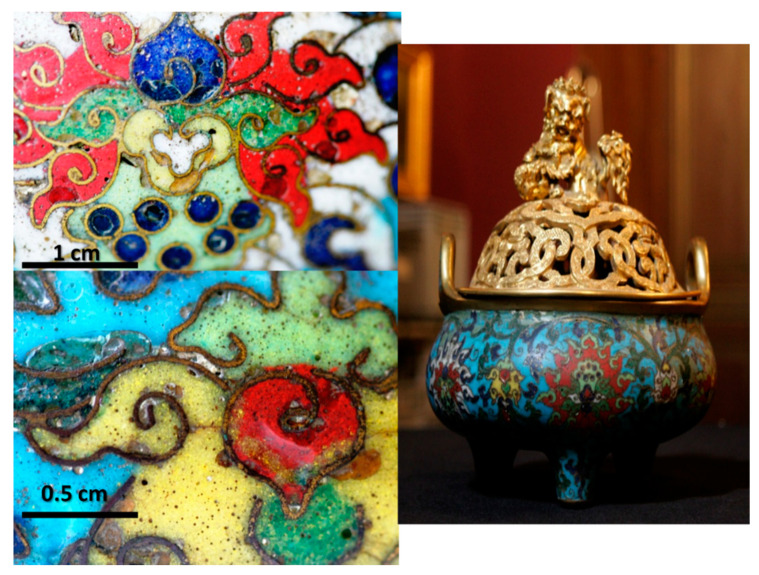
The incense burner (F1448C) and details of the *cloisonné* enameled décor (Kangxi reign). Note the many bubbles in the décor revealed by surface polishing.

The works of art brought to Paris by the French soldiers during the sack of the Summer Palace (*Yuanming Yuan*) in Beijing in October 1860 by the Anglo-French troops in response to the execution of European persons, as well as the gifts offered by the Embassy of Siam in June 1861, were the origin of the creation of the Chinese Museum in the castle of Fontainebleau. Empress Eugenie received a part of the objects collected in China, as did Queen Victoria of the United Kingdom [54,55,56]. The Empress, who appreciated the sunny exposure of the ground floor of the *Gros Pavillon* (designed by A.J. Gabriel) at the Château de Fontainebleau, near the carp pond and the English Garden, had asked A. Pacard, her architect, to set up a large living room for use during summer stays. The collection was open to rare visitors from 1863. The Museum was enriched at this time with other oriental objects, in particular personal gifts from the Emperor to his wife during the feast of Saint Eugenia. Photographic details are available [57,58,59]. The pair of ewers (Figure 1) and the basin (see [57,58,59]) are believed to have been made by the imperial workshops for the Summer Palace. They were acquired on the art market and offered by Napoleon III to the Empress as a birthday present. The *cloisonné* enameled incense burner (Figure 2) is also expected to have come from a Chinese Palace.

### 3.2. The Visual Characteristics of the Enamels

#### 3.2.1. Incense Burner (Assignment: Kangxi Period 1661–1722)

Figure 2 shows the incense tripod (inventory number F1448C, height: ~27 cm; diameter: ~26 cm, belonging to the collections of The National Museum of the Château de Fontainebleau (Chinese Museum)). The lower part is decorated with *cloisonné* enamels depicting multicolor lotus flowers and foliage scrolls characteristic of the influence of Buddhism on a turquoise background. The lid, rim and handles are gilded. The lid consists of golden interlacing on which a *Fô* female lion (*shi*) with a baby is placed at the top (the female lion is the symbol of education). The lid is expected to have been made by the lost wax technique, with subsequent chisel carving and gilding.

The three feet of the body are added with rivets visible inside. The handles appear to have been welded. The *cloisonné* enameled areas are turquoise, white, dark blue, red, green, light green and yellow. The center of the flowers is either yellow or green and these colors are used sparingly, which is consistent with their less availability or high cost. The use of different greens is significant. Numerous pores and cracks are visible in the enameled decoration (Figure 2). These defects are frequently encountered in such objects due to the application of *cloisonné* enameling. The *cloisonné* technique particularly involves the interposition of the glass powder between the metal sheets (‘*cloison*’ in French) fixed perpendicular to the object by point welding, glue or wax (before the filling of the voids in between with enamel powder) and then the firing step which requires heating/melting the enamels. The latter operation is repeated several times. This induces a significant shrinkage of the enamels, but the duration of the firing cycles is too limited to eliminate the bubbles. A surface polishing step is also performed at the end which enhances the open porosity.

#### 3.2.2. Ewer (3rd Quarter of 18th Century, Qianlong Mark)

Figure 1 shows the ewer analyzed (from a pair, inventory number F1467C; height: ~40 cm; base: ~27 × 20 cm^2^; weight ~2750 kg, belonging to the collections of the National Museum of the Château de Fontainebleau (Chinese Museum)). One of the ewers was specifically analyzed while a few measurements were also performed on the other one. The object is gold like its pair and the basin, which especially puts these objects in an exceptional category. However, the marks are different (Figure 1). The objects appear to have been made by shaping/hammering gold foil. Traces of welds are visible in a few places (handle, spout). The interior has a greenish counter-enamel. The ewer is decorated both with *cloisonné* and painted enamels. The medallions were formed in gold body before being painted and fired. As the *cloisonné* decoration requires several fillings with enamel powder between the firing steps followed by a final polishing, we can assume that its creation was done before decorating the medallions (or the medallions were prepared before to be welded on the gold frame).

**Figure 3:**
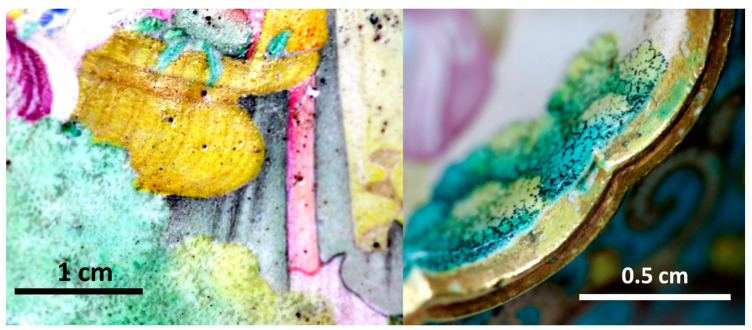
Detailed images of the enamels of the gold ewer décor (F1467C).

The *cloisonné* décors consist of multicolor flowers (blue, yellow, red and green) on turquoise and blue backgrounds. The background is turquoise for the belly as usual but blue for the foot and the neck. The quality of the *cloisonné* décor is also exceptional by its surface with less bubble (Figure 1) than in the Kangxi incense burner (Figure 2). The painted décors of the small medallions depict flowers, personages and/or landscapes (four medallions on the neck, six on the central part and four on the foot) [57,58] and one large medallion on each face depicts women with a child in a garden. The fineness of the design and the variety of color tones demonstrate great technical mastery. The decor style appears to be a hybrid, combining characteristics of Chinese decor (shape of the houses) and European decor (women’s faces, basket of flowers, belt and drape of the dress). The enamels are rather matt (Figure 1), except some yellow areas (Figure 3, this should involve a lower melting temperature of the enamel colored in yellow), giving the impression of a decoration on paper, which is unusual for an enamel decoration. The artist used the stamping of the metal support of the medallion for some part of the decoration (window of the pavilion, garden fence). Burrs and drips of the enameled decor on the edge of the medallions are observed (Figure 3).

## 4. Results and Discussion

### 4.1. Elemental Analysis

#### 4.1.1. Metal Body

**Figure 4:**
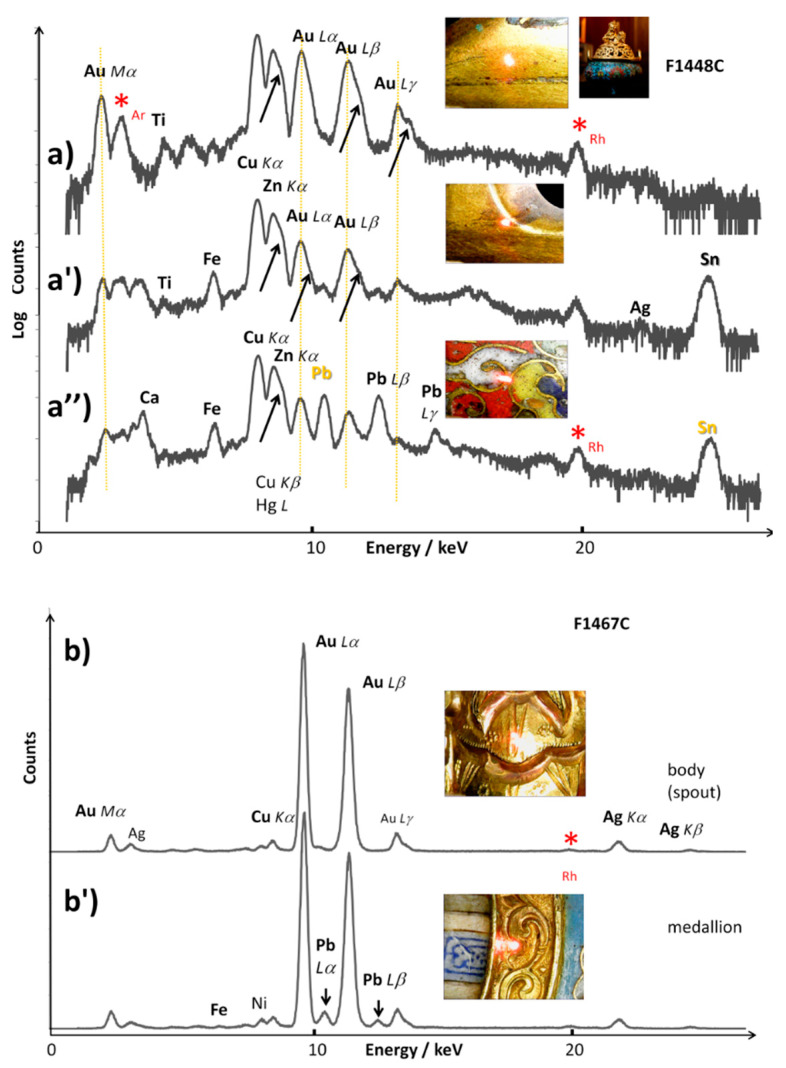
XRF spectra recorded on the metal parts of the incense burner (**a**,**a′**,**a″**) and the ewer (**b**,**b′**); the lightened spot on the photograph shows the analyzed area: (**a**) rim, (**a′**) rim with gilding partially lost and (**a″**) *cloison*; (**b**) spout and (**b′**) medallion rim. Main XRF peaks are labelled; arrows indicate the contribution of Hg L peaks in the spectra a, a′ and a″. Red stars (*) indicate the contribution of the instrument (Rh peaks). The logarithmic scale on the top spectra makes more visible the contribution of minor elements.

Figure 4 shows the XRF spectra recorded on different parts of the metal body of the two objects. The spectra recorded on the complex non-uniform areas are shown with logarithmic scale in order to make the contribution of minor components more visible.

The analyses show that the rim of the incense burner (lower part) and the *cloison* foils are made of brass alloy (Figure 4a,a′′). Comparison of a spot exhibiting a nice gold color (Figure 4a) with a spot that looks corroded or with gilding partially lost (Figure 4a′), shows a strong decrease of the intensity of the Au peaks, that is consistent with a gilded Cu-Zn brass (Table 1). Subtracting the ‘contaminated’ data due to the gilding, the composition is approximately copper (Cu) 65 wt%, zinc (Zn) 28 wt%, tin (Sn) 6 wt%, and iron (Fe) 1 wt%. The shoulders marked with arrows in Figure 4 indicate the presence of small Hg L peaks which probably result from the cold application of the gold foil using mercury as a coating agent. The measurement of the composition is likely contaminated with the contribution of the adjacent enamels rich in Pb and Ca (see further). Volatilization of lead oxide during the firing process followed by the polishing step should also have polluted the whole surface of the artefacts. However, incorporation of lead in the brass composition is also common for Chinese brass [41].

The comparison of the compositions measured on different spots shows a variation in the Cu/Zn ratio. The difference concerning the measurements is assigned to the poor precision of the method and/or local heterogeneity of the alloy. Tin is also observed to some extent, as in the case of the body. This is consistent with the commonly observed fabrication of Chinese brass which involves the bringing together of different types of brass pieces that do not have identical compositions [41].

The ewer body is made of a gold (84 wt%)–silver (14 wt%) alloy with a small addition of copper (1 wt%) (Figure 4b,b′). Copper increases the hardness and mechanical strength of the alloy while silver is also considered to promote the bonding with the silicate enamels [60]. The difference in the silver content (3 wt%) is assigned to the uncertainty of the method and to the contribution of lead contamination. However, the small amount of lead present is again assigned to the pollution of the metal surface provoked by volatilization and deposition of a lead oxide film on the whole surface from the enamels during the firing step. The highest value of lead was indeed measured close to the enameled areas.

#### 4.1.2. *Cloisonné* Enamels

The concept of ‘enamel composition’ is meaningful for a thick and homogeneous enamel like that of a transparent porcelain or celadon glaze. It remains relevant if the enamel is colored by ions, while it is much less when the coloring is done by pigments where the proportion varies according to the color. Therefore, the composition of the silicate matrix and the pigments should be distinguished. Painted enamels are formed of thin layers very loaded with pigment, only one or several types of pigments forming mixtures at sub-micron to micron scales (see later). The concept of ‘composition’ therefore no longer makes sense without specifying very precisely the color and volume of the material concerned (e.g., in the case of microdiffraction or microfluorescence on a homogeneous small volume). However, the depth probed by XRF varies according to the energy of the photons and therefore the element being measured. In addition, light elements such as sodium but also oxygen and boron are not measured in the case of pXRF. It is therefore only possible to consider the type of enamel, more precisely the nature of the fluxes detected by XRF, and in some cases their relative proportion. Measurements of alkali and earth-alkali elements correspond to the upper layers that can be corroded. Measurements are more accurate for transition metals, the in-depth penetration of XRF characteristic photons being in the order of the enamel thickness. On the other hand, the measurement of heavy elements (Pb, Sn, Sb) is ‘polluted’ by the contribution of the substrate [45,52,53].

**Figure 5:**
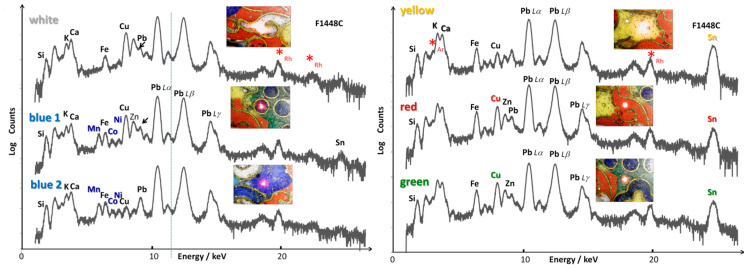
Representative pXRF spectra recorded on the different colored *cloisonné* enamels of the incense burner (white, blue, yellow, red and green areas). Red stars (*) indicate the contribution of the instrument (Rh peaks).

**Figure 6:**
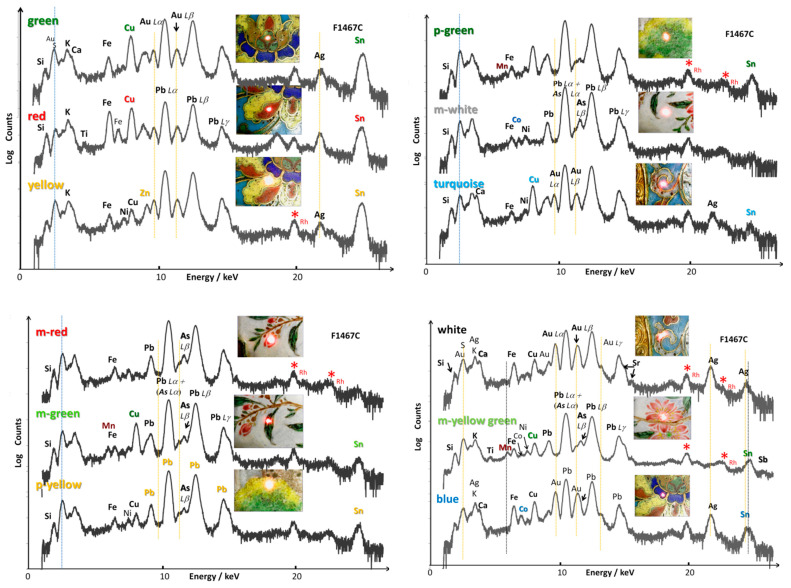
Representative pXRF spectra recorded on the different colored *cloisonné* (top-left: green, red and yellow; top-right: turquoise; bottom-right: white and blue) and painted (p-painted (large medallion) and m-(small) medallion) enamels of the gold ewer (white, yellow, red, green and yellow-green areas). Vertical lines serve as a guide for eyes to distinguish better the contribution of elements having peaks at very similar energy levels. Red stars (*) indicate the contribution of the instrument (Rh peaks).

Figure 5 and Figure 6 show the representative pXRF spectra recorded on the enamels of the Kangxi incense burner and the Qianlong ewer, respectively. The silicon, potassium, calcium and lead peaks arise from the glassy silicate matrix of the enamels. The iron peak is always observed, even in the white areas, and is related to the iron impurities commonly present in silicates. Transition metals were detected as chromophores such as cobalt in the blue area, and copper in the red, green and turquoise areas. Tin is also observed in many colored areas (yellow, red, green and blue).

Incense burner. We now consider the pXRF spectra recorded on the enamels of the incense burner in detail (Figure 5). The Ca peak appears to be stronger than that measured for *cloisonné* and painted enamels of the ewer (Figure 6). This is confirmed by comparing local ‘composition’ from the net count areas (Table 2). A very small amount of tin is found in the white area, but the amount is too small to contribute to the opacification. A small Co peak was detected in the blue area, much smaller than Mn and Fe peaks which are common for the blue enamel [42,45,52,53]. This finding is related to the use of Asian cobalt ores, rich in manganese [35,36,42,61,62,63,64,65,66]. It is here worthy to mention that the resolution of the instrument does not permit to separate the residual contribution of the Fe K_β_ peak that superimposes the Co K_α_ one, leading some inaccuracy in the measurement of the Co content. Cobalt is obvious in the spectrum only if the superimposed peaks of Co K_α_ and Fe K_β_ exhibit a stronger intensity than the relative intensity ratio of these peaks. Note the very high coloration power of Co^2+^ ions in the glassy matrix, showing that ~0.5 wt% of cobalt oxide is sufficient to obtain the dark blue color [62]. Consequently, the amount of cobalt is always found to be low to very low. On the contrary, more than 5 wt% of iron oxide is required to achieve the significant coloration of a silicate type of glass [62] and a certain level of iron (~2 wt%) does not color glassy silicates significantly, especially when firing is made under a reducing atmosphere. It is also very similar in the case of manganese. Comparison of the net count areas of K_α_ lines of Mn and Co is rather accurate and gives a ratio between 5 and ~20 (Table 2). In the blue 1 spot, the relatively high intensity of Cu and Sn peaks are due to the contribution of the adjacent green enameled area. However, the XRF spectrum of the blue 2 spot obtained from inside the larger enameled area with cobalt points out that copper is also present in the blue enamel. The addition can be voluntary, but some cobalt ores also contain copper [60].

In the yellow area, the presence of intense Pb and Sn peaks indicates the use of lead pyrochlore pigment (also called *Naples yellow*) [67,68,69,70,71,72,73,74]. The red area also shows the significant contribution of copper, indicating the ancient technique of red coloration in Chinese glass and glazes [75,76,77,78,79,80,81] where the red color is obtained by dispersion of Cu° nanoparticles. Additionally, the Fe peak seems to be greater than in the white and blue enamels, pointing out that it may have contributed to the red color in the form of hematite precipitation. The intensity of the Sn peak is rather strong, which may indicate that the yellow pigment had been mixed to adjust the hue or tin was used for the reduction of copper ions.

The Zn peak is also present but contamination of the measurement by the contribution of the *cloison* metal is certainly effective. The green enameled area shows a significant Cu peak, due to the use of Cu^2+^ ions in the silicate matrix for green coloration, the standard technique used to color an alkali-based glass turquoise.

Table 3 compares the typical oxide compositions of Chinese *cloisonné* and Limoges painted enamels taken from the literature [38,39]. It is observed that the dispersion of the data is large [39]. However, Chinese enamels are much richer in lead oxide (15 to 40 wt% PbO that corresponds to ~10 to 30 at% Pb) than alkali-based Limoges enamels (~2 to 13 wt% PbO). The composition of the glassy matrix of the *cloisonné* enamels of the incense burner shows some similarity with the low lead glazes, such as Limoges enamels, and as previously observed for rare 17th century Chinese *cloisonné* enamels [38,39].

Gold ewer. Regarding the ewer (Figure 6), Au (sometimes Ag) peaks are observed for all XRF spectra recorded on the *cloisonné* enamels due to the contribution of *cloisons* (and perhaps of the gold alloy substrate).

For the blue enameled area, cobalt is again found to be responsible for the blue color, as expected. However, it is significant that the blue *cloisonné* enamel does not exhibit the Mn peak, contrary to that measured on the incense burner. This indicates that a different source such as the imported European cobalt was used in the ewers. The higher intensity of the Kα line is consistent with the use of smalt, the potassium-based glass obtained by mixing with cobalt ores [62]. Observation of a low intensity Sn peak is consistent with the addition of tin to adjust the hue. The red enamel exhibits intense Cu and Sn peaks, as also observed for the incense burner. Tin is usually added to glass in order to promote the reduction of copper ions into Cu° nanoparticles at the origin of the red color production [82]. The high intensity of Fe peaks could also suggest the use of hematite to adjust the hue. Yellow and green enamels show significant Pb and Sn peaks, indicating the use of lead–tin pyrochlore pigment plus a Cu peak for the green enamel where the Cu^2+^ ions act as the coloring agent.

Table 2 and Table 4 compare the metal content (net count areas) of the blue, yellow and green enamels extracted from the fitting, respectively. These compositions are only comparative due to the intrinsic heterogeneity of the enamels, the contamination by neighboring phases and uncertainty of the method. The variable penetration depth as a function of the photon energy makes that the measured volume is very different as a function of the element [52,53,83,84]. Nevertheless, the comparison of measurements made on similar blue spots shows consistency, taking into account the contribution of neighboring phases.

**Figure 7:**
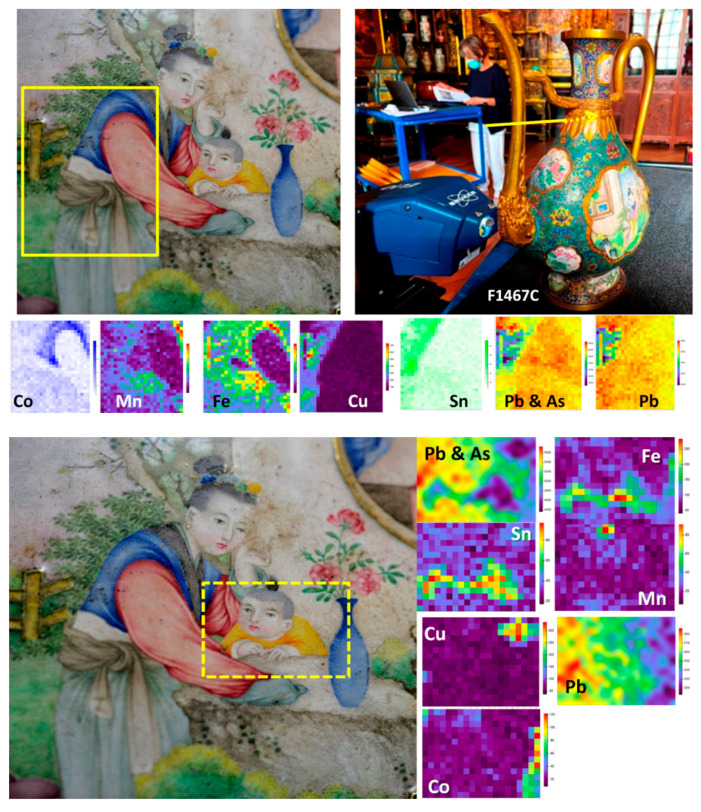

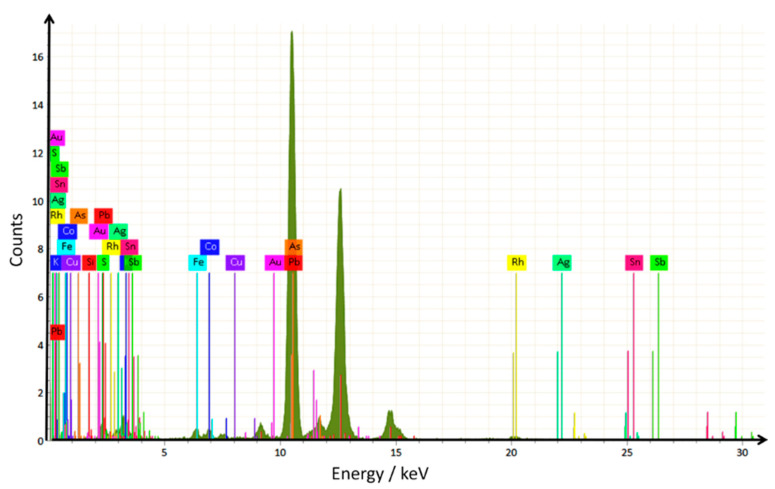
pXRF maps recorded on the painted décor depicting a woman with a child in the garden. The two mapped areas are delimited with a yellow line. The optical pixel image and corresponding distribution of elements (Co, Mn, Fe, Pb, Cu, Sn and Pb + As) are shown. An example of an XRF spectrum recorded on the darkest blue area of the woman’s vest in the painted décor.

The cobalt content of the blue enamel of the Kangxi incense burner is lower than that of the Qianlong ewer but the former is associated with manganese (Mn/Co net count area = 5 to ~20) while arsenic was measured for the latter and no significant amount of manganese was detected (Figure 5, Figure 6 and Figure 7). In some spots (Blue 2 spot, Table 2) a certain level of arsenic was also detected by XRF but the absence of a characteristic As-O Raman band indicates that As remains dissolved in the silicate network.

This indicates that some of the cobalt raw materials used contain some arsenic. Simultaneous use of cobalt ores from different origins has previously been reported [62]. These findings indicate the use of Asian Mn-rich cobalt in the blue enamel of the incense burner and European As- and Co-rich smalt for the similar enamel of the ewer [62], the potassium amount being much higher in the blue enamel of the ewer (K/Ca net count area ~1–2 for the incense burner vs. 8 for the ewer). In the ewer, as shown in Figure 6 and Figure 7, manganese was not detected in the blue areas. The Mn/Co net count area ratios comprised between 4 and 8 are typical of measurements made on blue-and-white porcelains of the Xuande period in the Ming Dynasty [62,64,65,66,85,86].

The compositions of the yellow and green *cloisonné* enamels of the incense burner are rather comparable (Table 4). The detection of cobalt in the green indicates that the color was obtained by adding a yellow pigment to the blue matrix, as was characteristic of the European method. Copper ions further contribute to the coloring process. On the other hand, the *cloisonné* enamels of the ewer contain much more lead (Pb/Si net count area ratio ~50 for incense burner vs. ~250 for ewer, except for lead-poor red enamel) than those of the incense burner and show similarity with the compositions of the painted enamels (a possible argument to support the hypothesis that the last firing is made for painted enamels but also to obtain a high gloss on polished enamels). Here it should be recalled that lead-rich compositions are common for Chinese *cloisonné* enamels (Table 2; [38,39,87,88]). They also contain a significant amount of tin, indicating the use of an opacifying agent to adjust the hue and/or as an underlayer on which the other colors are put. Copper was also measured in the green enamels at a high level.

#### 4.1.3. Painted Enamels (Ewer)

The most striking feature in the XRF spectra of the painted enamels deals with opacification based on the use of arsenic in the white enamel (Figure 6, top right and Table 4) and a small amount of antimony in the yellow-green enamel (Figure 6, bottom right) of the medallions. Cobalt was also detected as a whitening agent in the white areas opacified with arsenic. The As L_α_ peak cannot be separated from the Pb L_α_ one by the pXRF instrument and the As L_β_ peak is rather close to that of the Au L_β_. However, the As L_β_ peak is sufficiently well identified in some of the pXRF spectra such as m-red (painted medallion décor, Figure 6, left-bottom), m-green (painted medallion, Figure 6, left-bottom), p-yellow (painted large medallion depicting a woman and child, Figure 6, left-bottom) and m-yellow-green (Figure 6, right-bottom). Confirmation is given by the Raman analysis (see later). However, calculation of the elemental content is difficult, except for the (homogeneous) white enamel (Table 4) which is about 5 wt% of As_2_O_3_. Tin was also measured in the green, yellow and yellow-green painted areas (yellow fence on the left side and green leaves around). It is also important noting that tin was used for the white hand of the child and not for the face. This could indicate that the painting of the hand and the face had been made by different artists. The distribution of lead and arsenic is wide and not directly related to the drawing, especially for the child. This is consistent with the deposit of a white arsenic and lead-rich layer as a substrate in the whole décor. The variation of the intensity reflects the difference in the thickness of this layer in relation with the variable planarity of the gold foil substrate. The poor planarity of the gold foil was also observed by X-ray radiography. Comparison with the red cloth of the woman’s arm and the corresponding mapping of iron and copper elements demonstrate the absence of iron- or copper-based phases. The pink hue had thus been made with gold nanoparticles and not with the alternating techniques based on hematite or copper nanoparticles. The Mn-rich mapping spot of the child clearly corresponds to the hair bun. Manganese-free cobalt is also obvious for the blue vase.

Data measured on the similar areas are rather identical, which allows confidence in the comparison. Note that the lead content of the white area, that seems to serve as a substrate layer on which the other colors are painted, is lower than those of the colored areas. This is consistent with a deposit of the white layer first, perhaps with the preparation of *cloisonné* enamels (and their polishing). The colored décor is then added and fired at a temperature lower than the *cloisonné* enamels and painted white enamel layer, that impose a higher lead content in colored painted enamels. The highest lead content was measured for the yellow color, and we further see by Raman scattering that yellow color was obtained by lead–tin pyrochlore pigment (Pb_2_Sn_2_O_6_ *Naples yellow* type) which imposes a saturation of the glass matrix by lead to preserve the dissolution of the pigment in the flux. The higher lead content involves a lowering of the melting temperature and viscosity of yellow enamel, according to the higher gloss and the smooth surface of painted yellow areas (Figure 3).

The latest generation XRF mobile instruments make it possible, if the surface to be studied is flat, to carry out maps as in Figure 7. The mapping process takes longer total measurement times than the analysis of a single spot but makes it possible to ensure the representativeness of the point measurements. An example of the spectrum recorded for a few selected pixels (blue area) is shown in Figure 7 where a well-defined Co peak is present despite the rather limited counting time imposed by the mapping procedure. In the same Figure, the comparison of the signal intensities of the woman’s blue vest relating to cobalt and manganese clearly shows that the correlation between them excludes the use of cobalt ores from Asian sites used under the Ming Dynasty [62,64,65,66,86], and supports the use of cobalt imported from Europe (smalt), also deduced from spot analysis. Note that manganese was used in the brown belt of the woman.

The distribution of tin in the green zone is obvious, as is its non-use for the white belt obtained by an arsenate. Addition of a little copper in the green areas is obvious. The black eyes of the child were obtained with an iron-rich compound (likely a spinel). The hands are colored in white with tin (cassiterite) although the face is made with arsenic and lead (lead arsenate).

### 4.2. Phase Raman Identification

**Figure 8:**
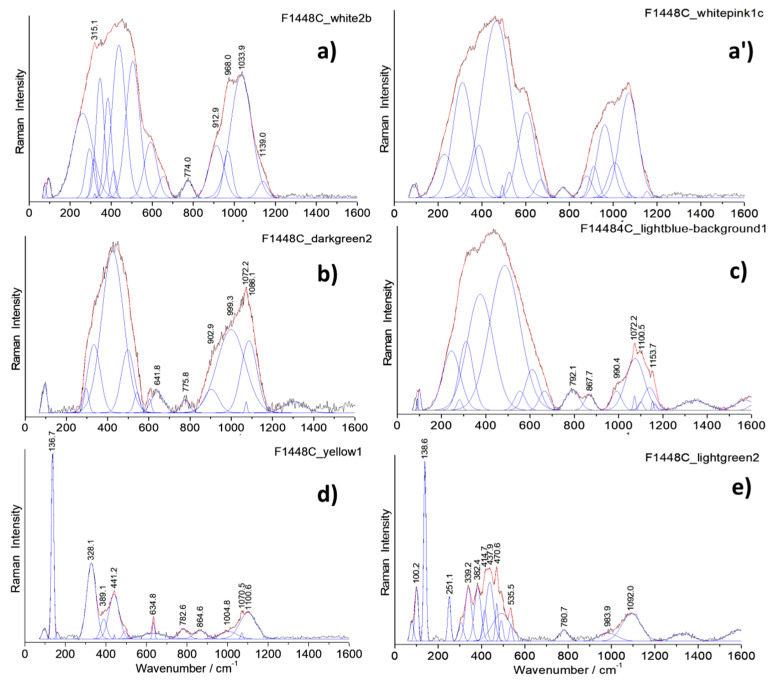
Representative Raman spectra recorded on the *cloisonné* enamels of the incense burner (baseline subtracted): white (**a**), white-pink (**a′**), dark green (**b**), turquoise (**c**), yellow (**d**) and light green (**e**) areas. Gaussian and Lorentzian components used to separate the contribution of the crystalline pigment from that of the glassy matrix are shown.

**Figure 9:**
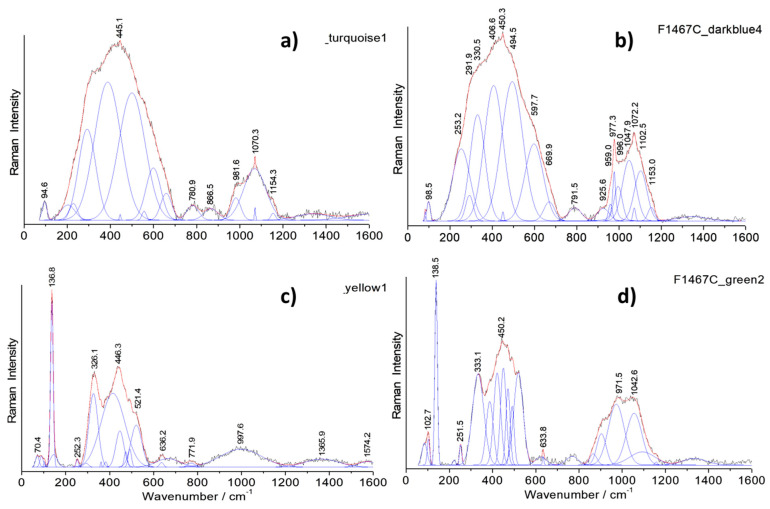
Representative Raman spectra recorded on the *cloisonné* enamels of the ewer (baseline subtracted): turquoise **(a)**, dark blue (**b**), yellow (**c**), and green (**d**). Gaussian and Lorentzian components used to separate the contribution of the crystalline pigment from that of the glassy matrix are shown.

**Figure 10:**
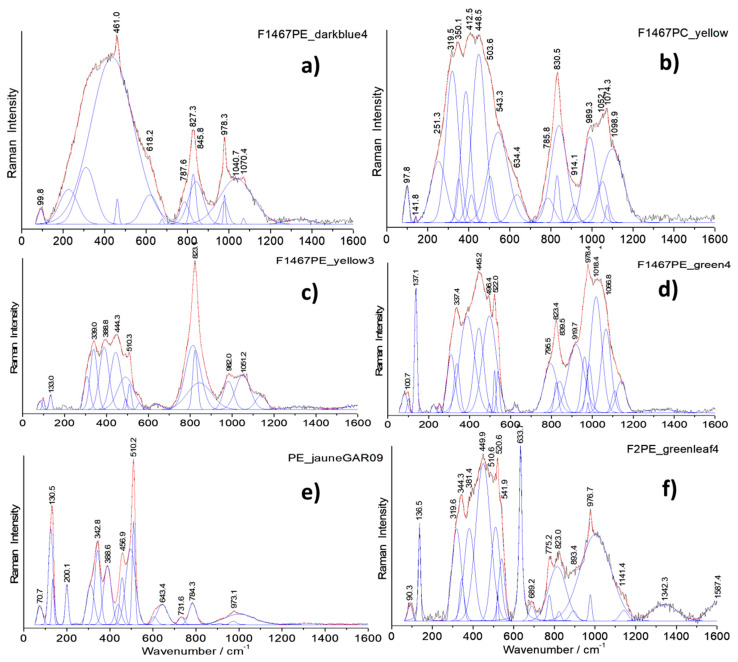
Representative Raman spectra recorded on the painted enamels of the ewer (baseline subtracted): dark blue (**a**), yellow (**b**,**c**,**e**), and green (**d**,**f**).

Figure 8, Figure 9 and Figure 10 show representative spectra recorded with a 200× long working distance (lwd) microscope objective. Preliminary Raman identification made using a lower magnification microscope objective (50× lwd) was published in ref [52] in the course of the study of a series of Chinese enameled wares. Figure 8 and Figure 9 show representative spectra of *cloisonné* enamels while Figure 10 shows those of painted enamels. Characteristic peak wavenumbers and phase assignments are summarized in Table 5. The Raman spectra obtained from the different colored enamels of the F1448C incense burner and F1467C ewer were baseline subtracted and spectral components were then specified with a peak-fitting process to identify the crystalline phases more clearly.

#### 4.2.1. Silicate Matrix and Crystalline Phases

The *cloisonné* and painted enamels studied display the typical Raman signature of a glassy silicate, sometimes accompanied by some crystalline phases either coming from the raw materials used or from the addition of pigments. The Raman spectrum of a glassy silicate is mainly dominated by two broad ‘bands’ at about 500 and 1000 cm^−1^, arising from the bending and stretching modes of the SiO_4_ tetrahedron which polymerizes to form the crystalline or amorphous silicates [47,48,49,50,51]. The Si-O connectivity is interrupted with other elements (Al, K, Pb, etc.) coming from the fluxes used in the glass raw materials. In the glassy silicate signature, the symmetrical stretching mode of the SiO_4_ vibrational unit dominates the Raman spectrum and the contribution of the Al-O bond (too ionic) is very poor and that of the Pb-O located at low wavenumber is suppressed by the baseline subtraction [37]. Therefore, the SiO_4_ stretching mode provides a direct link between its spectral components and the SiO_4_ tetrahedron with different connectivity. In this case, five components are present due to the contribution of isolated tetrahedron, and of tetrahedron connected by one, two, three or four common oxygen atoms, forming the glassy polymerized Si-O network [47,48,49,50,51]. Consequently, the 700–1250 cm^−1^ wavenumber range is fitted by five components. Additional bands also arise from the contribution of crystalline phases. The number of components in the bending ‘band’ is much higher (the symmetrical bending mode of a SiO_4_ tetrahedron has E character and the asymmetrical mode F character that could generate 25 components) and it is not possible to assign a physical meaning to the components of the fitting.

The quality of the spectrum collected with mobile instruments where the Rayleigh scattering is rejected by only one edge filter is less than that recorded with advanced fixed instruments equipped with a set of filters. In particular, the background of the mobile instrument is not flat, which makes its baseline subtraction partly subjective. Consequently, the fitting of the spectrum is affected by the intensity of the background and the set of SiO_4_ stretching components should be considered mainly as a tool to determine the contribution of crystalline phases precisely.

Raman spectra recorded on white, white-pink, dark green and light blue *cloisonné* enamels of the incense burner (Figure 8) and of the turquoise and dark blue *cloisonné* enamel of the ewer (Figure 9) reveals mainly the signature of the glassy (lead earth-alkali-based) silicate: the center of gravity (and roughly the maximum) of the stretching band peaks at 1030–1050 cm^−1^. For painted enamels (ewer) the stretching massif peaks at a slightly lower wavenumber, ~1000–1030 cm^−1^, which indicates a more depolymerized silicate matrix, according to the higher content of lead measured (Table 3 and Table 4). This should indicate a firing at a lower temperature, according to the production sequence assumed above. Small narrow peaks are, however, observed at ~975 and 1070 cm^−1^ which are assigned to alpha- and beta-wollastonite (CaSiO_3_) precipitates, according to the large amount of calcium (Table 4). Painted enamels (Figure 10) show the similar Raman signature of the glassy silicate matrix for white and blue areas (bending and stretching broad bands) plus some additional features, such as the 461 cm^−1^ peak of alpha-quartz. This peak seems to be more frequent in the painted enamels than in the *cloisonné* ones, indicating different preparation routes.

#### 4.2.2. Pigments and Opacifiers

Raman analysis is particularly effective in the identification of crystal phases present in the glassy silicate matrix. In the white *cloisonné* enamel of the incense burner, the weak peak at 315 cm^−1^ is assigned to fluorite (CaF_2_) as an opacifier which specifically belongs to an ancient Chinese tradition of glass opacification [38,52,81,88]. The continuous use of fluorite in Chinese glassy materials has been reported from the Tang Dynasty to the Qing Dynasty, in glass objects [81] as well as *cloisonné* enamels [38,87,88,89].

Regarding the blue enamels with different hues, different spectral features were observed in the artefacts studied. In the light blue-turquoise background *cloisonné* enamel of the incense burner, the typical bending and stretching bands of the glassy silicate structure are observed along with a small band at 868 cm^−1^ which can be attributed to the stretching mode of the chromate phase. This phase probably results from the presence of chromium often associated with the cobalt ore used [62,90]. The Raman spectrum of the dark blue *cloisonné* enamel of the ewer displays only the signature of a glassy silicate, indicating the use of Co^2+^ ions dispersed in the glassy matrix to obtain the dark blue color, without the precipitation of any crystalline phases. On the contrary, the painted blue enamel of the ewer strikingly shows a distinctive ~820 cm^−1^ peak with a shoulder at 788 cm^−1^ (Figure 10a,b,d) which is characteristic of the As-O symmetrical stretching mode in a lead arsenate phase. This feature is particularly assigned to lead–potassium–calcium arsenate with an apatite structure [52,53,61,63,74,91] which is formed by the reaction of lead, potassium and calcium coming from the enamel matrix with arsenic coming from the cobalt source [10,46,62,74]. In some cases, the As-O mode is of a larger intensity (Figure 10c), which indicates very small apatite grains or the formation of another kind of As-based phase (As-feldspar?) [62]. The Raman spectra recorded on red *cloisonné* enamels do not show peaks characteristic of any crystalline phases (see ref. [52]). This is consistent with coloration with copper nanoparticles.

Raman spectra recorded on the yellow and green *cloisonné* (Figure 8 and Figure 9) and painted enamels (Figure 10) mainly consist of a set of narrow bands, with a strong peak at ~130 cm^−1^ characteristic of lead-based pyrochlore pigment (also called *Naples yellow*) [67,68,69,70,71,72,73,74]. This mode involving Pb atoms peaks at low energy due to their heavy mass. *Naples yellow* can now be considered as a general pigment class, based on lead, antimony and/or tin (*Naples yellow* type I) with varying stoichiometry due to different routes in the production process. The pyrochlore structure may incorporate different ratios of these elements along with others such as iron, zinc and silicon (*Naples yellow* type II), forming complex solid solutions depending on the oxygen stoichiometry (i.e., the degree of oxidizing/reducing atmosphere in the firing). The availability of the raw materials and the desire to achieve different hues result in the modification of the pigment which is further affected by the glaze raw materials during the firing process. At least three types of lead pyrochlore pigment were identified in the objects according to the Raman spectra: The end member Pb_2_Sn_2_O_6_ type (*Naples yellow* type I in the literature) in the yellow *cloisonné* enamels of the incense burner (Figure 8d) and the ewer (Figure 9c); a second tin-rich phase in the light green *cloisonné* enamel of the incense burner (Figure 8e) and green *cloisonné* enamel of the ewer (Figure 9d); and the tin-antimony-(zinc?) pyrochlore phase in the yellow painted enamel of the ewer (Figure 10e) as well as its green painted enamel (Figure 10f). The first type of lead pyrochlore Pb-Sn pigment is characterized by the strongest peak at 137 cm^−1^ and distinct ~330 and ~450 cm^−1^ components. The latter component is particularly assigned to the stretching mode of Sn-O (*Naples yellow* type I). The second type of pyrochlore pigment as the tin-rich phase has further components at 382 and 471 cm^−1^ along with a characteristic ~250 cm^−1^ peak while the third type as the mixed pyrochlore phase notably displays a ~510 cm^−1^ strong component which belongs to the Sb-O stretching mode. In the Raman spectra of lead pyrochlore pigment, the evident ~ca. 135 cm^−1^ peak characteristic of Pb-O mode is due to the saturation of the glassy silicate matrix with excess lead. Its position depends on the firing temperature employed [67,68,69]. The results are consistent with the XRF measurements. The Sn peak is clearly observed in all yellow to green areas (Figure 5 and Figure 6, Table 4). The Sb peak was only detected for yellow-green painted medallion areas (Figure 6) as well as *cloisonné* enamels (Table 4).

Other stringent features are the narrow peak doublet at ~633 and ~775 cm^−1^ characteristic of cassiterite (SnO_2_) [39], particularly in the case of the green painted enamel of the ewer (Figure 10f). In some of the yellow and green painted enamels analyzed, the signature of lead arsenate apatite phase is also observed (Figure 10b,c,d,e). In some of these spectra, the As-O mode is larger (Figure 10b,c), which may indicate very small lead arsenate apatite grains or the formation of another As-based phase (As-feldspar?) [62].

### 4.3. Painting Technique

**Figure 11:**
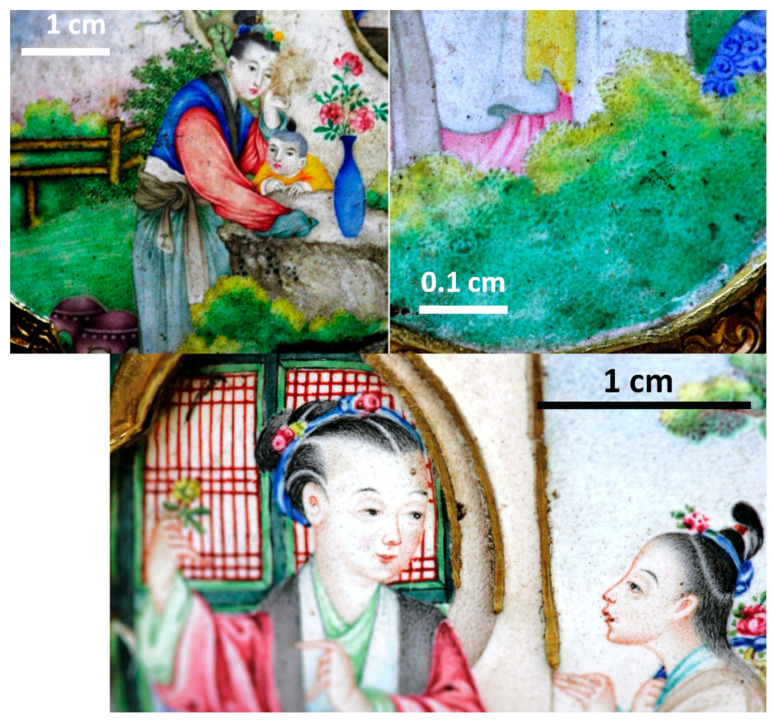
Images of the painted décor and zoom of the green foliage showing the use of dots of different hues and different painting techniques (spitting or brush touch).

The observation of the painting technique of the Qianlong ewer at high optical magnification (Figure 11) shows dotted touches of color, which is the technique of the miniaturists in the 18th century [92,93]. Miniaturists made drawing and painting at a small scale (a few cm^2^) representing landscapes or scenes including many personages, country or castle views, etc. They used lenses, fine nibs and brushes made of some polishes to achieve these miniature decorations. It is reasonable to think that similar techniques were used to paint the decoration of the enameled ewer. Only for some parts such as the hair of the human figures, the brush touch was used. The analysis of a fraction of a green paint touch is shown in Figure 10f. Here, the spot analyzed in Raman with the 200× objective is about more than ten times smaller than the paint point visible in the zoom image (Figure 11). However, in this spot at least four crystalline phases (cassiterite, lead pyrochlore type 3, wollastonite, lead arsenate apatite plus amorphous carbon) and the amorphous silicate matrix are identified in the Raman spectrum. This confirms the use of a color palette prepared by prior mixing of coloring agents to obtain a wide range of colors, as practiced in European easel paintings, in accordance with the archival texts which say that about thirty colors became available for painted enamel decorations prepared by the imperial workshops [6,94,95].

## 5. Conclusions

XRF and Raman analyses enabled us to obtain a great deal of information about the coloring agents used in the different types of enamels studied as well as their glass types, despite the on-site analysis conditions with limited access time to the objects and the imperative use of non-invasive methods. The exceptional character of these objects in terms of their aesthetic quality is also attested in terms of their enameling techniques. The sparse use of new colors, such as yellow and green in the incense burner, is in perfect harmony with the imported origin of the recipes (complex lead pyrochlore type pigments, a simple lead–tin pigment being already used at least from the Ming Dynasty) used to create these colored enamels at the end of the 17th century or at the turn of the 17th–18th century. The observation of the same wavenumber at ~135 cm^−1^ for the pyrochlore pigment based on Pb-Sn in the yellow *cloisonné* enamels of the two objects and painted enamels of the ewer indicates the same temperature of preparation of the pigment, prepared before, probably around 600–700 °C which is compatible with the expected <800 °C according to the literature [20].

The complexity and mastery of the enamel decoration of the 18th century ewer as well as the extent of the color palette (Table 5) shows that the imported techniques were perfectly incorporated into the knowledge of the craftsmen of the Imperial Palace. It is evident that the lead content of the painted enamels is higher than that of the *cloisonné* ones for the ewer and much higher than that of the *cloisonné* enamels of the incense burner, except for the white background. This could also indicate that some of the recipes used in the incense burner had followed the European recipes introduced by the Jesuits. The increased XRF signal of potassium concomitant with the cobalt signal in painted enamels is a good indication of the use of smalt as a source of ‘European’ cobalt. This agrees with the results obtained for Japanese porcelain [18,19,20] as well as for paintings in China [94] and Japan [20]. The meticulousness of the dotted painting technique deposited on one or more backgrounds induces a complex stratigraphy of the enameled decoration. Although the sub-micron spatial resolution of Raman analysis allows access to grain-by-grain analyses, this is generally incompatible on-site because it takes too much measurement time to obtain such information. The lower resolution of the XRF analysis averages the related compositional data. In this case, only the availability of fragments (fragments collected during the restoration operation, sampling, shards from archaeological excavations) can lead to a more precise analysis for a better understanding of the stratigraphy of the enameled decoration in terms of composition. It is also necessary to compare the results of the analyses undertaken with the information found in the historical texts.

In conclusion, many questions still remain open. One of them is related to the functioning of the glass production workshop directed by the German Jesuit Kilian Stumpf. This workshop prepared the enamels, probably the frit, and enameled glass objects seem to have been produced at the beginning, painted enamels on copper or porcelain being produced after 1716 [95,96,97,98]. This could explain the privileged use of the opacification of lead enamels by the addition of arsenic, a classic technique of Italian glassmakers in the 17th century [99]. The use of a competing technique of opacification with cassiterite preferred by potters [99] appears to be very limited. The use of ‘Italian’ recipes (arsenic-based opacification, arsenic-based preparation of colloidal gold) could be linked with the venue of Italian coadjutor brothers with some expertise in the enameling techniques [100]. The highlighting of the use of European recipes in the *cloisonné* ware of the Kangxi period could indicate that the first attempts to use these European recipes were made for this type of object, as also for the ‘simple’ water pots as already observed [10,101]. It is necessary to analyze in detail a larger number of objects to statistically assess the use of imported recipes and their adaptation by Chinese artisans. The present study demonstrates that several phases of lead arsenate had been used for opacification, one of them being apatite. Different explanations are possible, such as that the source of arsenic-rich cobalt is different. The other one could be that the compositions of the silicate matrix are different and hence different phases were formed. Furthermore, a combination of the two phenomena is also possible. It is very likely that different pyrochlores were used simultaneously for yellows and greens (two being rich in tin, another containing antimony, plus zinc). Only µdiffraction or transmission electron microscopy analyses can provide more confident answers, but these methods require sampling.

Detailed XRF mapping of the painted area should be correlated to stylistic study. The Sn element map shows very limited use of SnO_2_ for the child’s hand and not for the face. This could indicate that different artists contributed to the painting of the different parts of the décor.

The present study reveals the potential of on-site non-invasive studies but also the limitations of the method.

## Figures and Tables

**Table 1:** Composition of the metal parts of the objects studied (metal wt%).

Object	Part	% Au	% Ag	% Cu	% Zn	Cu/Zn	% Ti	% Sn	% Fe	% Pb
Incense burner	Gilded body	52	-	32	15	2	1	-	-	~1
	Body (gilding lost)	17	-	52.5	22	2.5	-	7	0.5	1
	Cloison	46	-	27.5	14.5	2	-	3	0.5	~1
Ewer	Spout	84	14	1	-		-	-	-	1.5
	Medallion rim	83	11	1	-		-	-	0.5	4

**Table 2:** Elemental (local) net count areas of characteristic peaks for the blue *cloisonné* enameled areas of the incense burner (IB) and ewer (E). Ratios calculated from the net count area. Ions contributing to coloration are in bold (- not calculated). The error deduced from the study of similar heterogeneous objects is less than 15% for major elements but could be 100% for traces.

Elements (Peak)	Blue 1 IB	Blue 2 IB	Blue Flower E
Si (Kα)	889	2244	900
Pb (Lβ)	136,403	150,608	103,673
**Pb/Si**	**153**	**23**	**115**
**Pb/K**	**67**	**26**	**15**
Sn (Kα)	1895	3571	1767
K (Kα)	2010	5841	6902
Ca (Kα)	2348	3032	814
**K/Ca**	**1**	**2**	**8**
Co (Kα)	**385**	**170**	**1591**
Mn (Kα)	**1895**	**2864**	**162**
**Mn/Co**	**5**	**17**	**0.1**
Fe (Kα)	2377	2516	7182
Cu (Kα)	**18,282**	1429	9924
Zn (Kα)	**8885**	260	204
As (Kβ)	-	**1770**	**4160**
**As/Co**	**~0**	**10**	3
Au (Lα)		364	31,030

**Table 3:** Comparison of the composition range (oxide wt%) of Chinese *cloisonné* and Limoges enamels (from Ref. [38]).

Oxide	China	China	Limoges
	16th	17th	17th–18th
SiO_2_	40–50	45–60	60
PbO	30–40	15–22	2–13
CaO	2–7	5–15	3–5
K_2_O	5–12	5–10	5
Na_2_O	0.5–15	0.3–15	-

**Table 4:** Elemental (local) near net count of characteristic peaks for the enameled yellow, green, red and white *cloisonné* and painted areas of the Kangxi incense burner (IB) and Qianlong ewer (E). Ratios calculated from the net count area. Elements contributing to coloration are in bold: C: *cloisonné* enamel; P: painted enamels, large medallion; m-: small medallion.

Elements	Yellow IB C	Green IB C	Yellow E C	Green E C	Red E C	Turquoise E C	White E C	m-Yellow-Green E P	m-Flower Green E P	p-Yellow E P	p-Green E P	m-White E P	m-Red E P
Si (Kα)	1793	1729	520	634	1608	1735	1245	1091	714	719	967	1141	1057
Pb(Lβ)	99,145	113,401	157,048	149,094	47,567	149,048	128,098	156,852	141,359	**169,959**	157,308	147,672	**161,192**
**Pb/Si**	55	66	302	235	3	86	10	144	198	236	162	129	152
Sn(Kα)	**12,003**	4395	**14,411**	**9352**	**9017**	644	400	1137	967	**1987**	1497	320	253
K(Kα)	**14,919**	**11,607**	5368	3893	5353	6144	4636	4865	4107	3790	3814	6388	5124
Ca(Kα)	272	278	5	327	239	**1859**	**1588**	1	46	10	78	1	2
**K/Ca**	55	42	1074	12	22	3	3	4865	90	379	48	6388	2562
**Pb/K**	7	10	29	38	5	24	27	38	34	45	41	23	3
Sb(Lα)	**6617**	3845	766	-	-	-	-	436	-			-	
Mn(Kα)	102	-	153	141	142	145	-	1074 *	706	191	353	-	73
**Mn/Co**	**0.6**	**0**	**1.1**	3.7	0.1	1.9	0	7 *	5	**	**	0	**
Fe(Kα)	3809	6390	1810	3232	17506	792	4343	1010	1424	1168		783	**1627**
Cu(Kα)	2111	**13,175**	3253	**25,331**	**26,120**	**17,790**	**5043**	**17,856**	**3847**	2131	**13,823**	73	212
Zn (Kα)	1161	6632	524	658	909	270	614	-	215	31	639	129	20
Co(Kα)	**180**	**428**	**142**	38	**1086**	77	**146**	153	**138**	32	27	**258**	16
Au(Lα)	-	-	7506	3969	2762	5797	26,640	-	-	-	328	-	-
Ag(Kα)	-	-		1860	-	-	5133	-	-	-		-	-
As(Kβ)	-	-			-	-	-	7668	8473	8073	6296	**10,511**	**10,392**

- Not included in the fitting; * addition of brown; ** Co value too small to be significant.

**Table 5:** XRF and Raman results of the different colored enamels studied (sh: shoulder, w: weak, m: medium, S: strong, vS: very strong).

Enamel Color	Enamel Type	Artefact Period	Major Element (XRF)	Minor/Traces Element (XRF)	Pigment Raman Bands (cm^−1^)	Phases	European Recipe/ Ingredient
white	*cloisonné*	Kangxi	Si,K,Ca,Pb	Fe,Cu	315 (w)	Fluorite	No
	*cloisonné*	Qianlong		Fe,Cu	-		
	painted			As,Fe,Ni,Sn	-		Yes
blue	*cloisonné* *(light blue)*	Kangxi		As,Mn,Fe, Co,Ni,Cu,Sn	868 (w)	Chromate	No
	*cloisonné* *(dark blue)*	Qianlong		Fe,Co,Cu,(Sn?,As?)	-	Glassy silicate matrix	No?
	painted			Co,Fe,As	788 (sh), 827 (S)	Lead arsenate apatite	Yes
yellow	*cloisonné*	Kangxi		Sn,Fe,(Cu?)	137 (vS), 328 (S), 441 (m) 635 (w), 783 (w)	Pyrochlore type I (Pb_2_Sn_2_O_6_) Cassiterite	Yes
	*cloisonné*	Qianlong		Sn,Fe,Ni	137 (vS), 326 (S), 446 (S), 521 (m) 636 (w)	Pyrochlore type 1 (Pb_2_Sn_2_O_6_) Cassiterite	Yes
	painted			Sn,Fe,Cu,Ni,As	130 (vS), 200 (m), 343 (S), 389 (m), 457 (m), 510 (vS)	Pyrochlore type 3	Yes
yellow-green, light green	*cloisonné*	Kangxi			139 (vS), 225, 251 (m), 339 (m), ~420 (m), 471 (m), 535 (w)	Pyrochlore type 2 (~250 cm^−1^)	Yes
	painted	Qianlong		As,Sn,Cu,Mn,Ni(Sb?)	[130–510]	Pyrochlore 3	Yes
green	*cloisonné*	Kangxi		Cu,Fe,Sn			
	*cloisonné*	Qianlong		Cu,Sn,Fe	138 (vS), 251 (w), 333 (S), 450 (S),510 (S)	Pyrochlore type 2	Yes
	painted			As,Cu,Sn, Fe,Cu,Mn	775 (m), 823 (m) 137 (S), 344 (vS), 450 (vS), 521 (vS) 634 (vS), 775 (m)	Lead arsenate apatite Pyrochlore type 3 Cassiterite	Yes
turquoise	*cloisonné*	Qianlong		Cu,Fe,Ni	-	Glassy silicate matrix	No
red	*cloisonné*	Kangxi		Cu,Fe,(Au?)			No?
	*cloisonné*	Qianlong		Fe,Cu,Sn			?
	painted			Au,Fe,Ni,As,			Yes

## Data Availability

All data incorporated in the paper.
